# Zooprophylaxis as a control strategy for malaria caused by the vector *Anopheles arabiensis* (Diptera: Culicidae): a systematic review

**DOI:** 10.1186/s40249-017-0366-3

**Published:** 2017-10-25

**Authors:** Abebe Asale, Luc Duchateau, Brecht Devleesschauwer, Gerdien Huisman, Delenasaw Yewhalaw

**Affiliations:** 1 Department of Biology, College of Natural Sciences, Jimma University, Jimma, Ethiopia; 2 Department of Animal Physiology and Biometry, Faculty of Veterinary Medicine, Ghent University, Ghent, Belgium; 3 Department of Medical Laboratory Sciences and Pathology, College of Health Sciences, Jimma University, Jimma, Ethiopia

**Keywords:** Malaria, Cattle, Mosquito, Vector control, Ethiopia

## Abstract

**Background:**

Zooprophylaxis is the use of wild or domestic animals, which are not the reservoir host of a given disease, to divert the blood-seeking malaria vectors from human hosts. In this paper, we systematically reviewed zooprophylaxis to assess its efficacy as a malaria control strategy and to evaluate the possible methods of its application.

**Methods:**

The electronic databases, PubMed Central®, Web of Science, Science direct, and African Journals Online were searched using the key terms: “zooprophylaxis” or “cattle and malaria”, and reports published between January 1995 and March 2016 were considered. Thirty-four reports on zooprophylaxis were retained for the systematic review.

**Results:**

It was determined that *Anopheles arabiensis* is an opportunistic feeder. It has a strong preference for cattle odour when compared to human odour, but feeds on both hosts. Its feeding behaviour depends on the available hosts, varying from endophilic and endophagic to exophilic and exophagic. There are three essential factors for zooprophylaxis to be effective in practice: a zoophilic and exophilic vector, habitat separation between human and host animal quarters, and augmenting zooprophylaxis with insecticide treatment of animals or co-intervention of long-lasting insecticide-treated nets and/or indoor residual spraying. Passive zooprophylaxis can be applied only in malaria vector control if cattle and human dwellings are separated in order to avoid the problem of zoopotentiation.

**Conclusions:**

The outcomes of using zooprophylaxis as a malaria control strategy varied across locations. It is therefore advised to conduct a site-specific evaluation of its effectiveness in vector control before implementing zooprophylaxis as the behaviour of *Anopheles arabiensis* mosquitoes varies across localities and circumstances.

**Electronic supplementary material:**

The online version of this article (10.1186/s40249-017-0366-3) contains supplementary material, which is available to authorized users.

## Multilingual abstract

Please see Additional file 1 for translations of the abstract into the six official working languages of the United Nations.

## Introduction

Humans have known malaria for thousands of years. According to the World Malaria Report 2016 [1, 2], there were an estimated 212 million cases and 429,000 deaths due to malaria in 2015, approximately 88% of which were in the African region. Similarly, most of the deaths (90%) also occurred in the World Health Organization (WHO) African Region; of these approximately 74% were children under 5 years of age. The incidence and death of malaria, however, was reduced by 21% and 29%, respectively, in 2015 worldwide in comparison to the situation in 2010 [1, 2].

Africa is the most affected region due to a combination of factors including the presence of very efficient malaria vectors (*Anopheles gambiae* sensu *lato* and *An. funestus*) and the predominant parasite species *Plasmodium falciparum*, which is the species mostly responsible for severe malaria [2]. Weather conditions, which often allow transmission to occur year round, scarce resources, and socioeconomic instability, which has hindered efficient malaria control activities, have also led to a high malaria incidence in this region [1, 2].

Malaria parasites are one of the first pathogens to be studied in a public health context due to the high level of morbidity and mortality in humans. There are four known species of *Plasmodium,* which cause human malaria, with a fifth added to the list most recently from the forested regions of Southeast Asia. These are: *P. falciparum*, *P. vivax*, *P. malariae*, *P. ovale*, and *P. knowlesi* [3, 4]*. Plasmodium falciparum* is the most virulent member of the group and it is responsible for the majority (99%) of malaria-related mortality [1, 3, 5]. The different *Plasmodium* species are host-specific though there have been periodic reports of simian malaria parasites being found in humans [4, 5].

The disease spreads from one person to another via the bite of a female mosquito of the genus *Anopheles* [5]. *Anopheles* mosquitoes belong to the order Diptera, family Culicidae, genus *Anopheles*, and series Pyretophorus. There are 465 to 474 described *Anopheles* species with 70 of its members recognized to transmit the *Plasmodium* parasite to humans [6]. Some of the species are species complexes because of the presence of morphologically indistinguishable sibling species within the complex [6]. For instance, the *An. gambiae* complex is a species complex composed of sibling species that are all difficult to identify morphologically using a taxonomic key but can be identified into its eight member species, namely *An. arabiensis*, *An. gambiae*, *An. coluzzii*, *An. merus*, *An. melas*, *An. bwambae*, *An. quadriannulatus*, and *An. amharicus*, using molecular techniques [7–9]*.*

*Anopheles arabiensis*, the subject of this review, is mainly found in subtropical and tropical savannah regions on the African continent. Its population distribution ranges from the western coast of Africa above the equator, to farther north into the Sahel, to the southwestern corner of the Arabian Peninsula, Sudan, Ethiopia, Kenya, Somalia, along the east coast, including Madagascar, and south into the desert and steppe environments of Namibia and Botswana in Southern Africa [6]. The adult *An. arabiensis* is well adapted to dry and forest sparse environments [10], whereas its immature stage prefers short-lived, sunlit, clear, and shallow aquatic breeding habitats mainly created by rainfall and human activities [11]. The density of larvae increases as the rainy season progresses. The abundance and development of the larvae is dependent on different physicochemical and biological factors [11], water turbidity and algae [12, 13], the presence of ammonium sulfate fertilizers [14], thermal limit [15], and the presence of maize pollen [16, 17].

In the eastern and southeastern African region where *An. arabiensis* remains the primary malaria vector, its population dynamics vary according to season, with its maximum population density recorded in the long rainy season from June to August [18]. It survives extreme dry seasons in the form of embryo dormancy in moist soil [19], continues reproduction using artificial breeding pans, and its population quickly builds up the following rainy season due to temporary breeding habitats being established [20].

The resting behaviour of *An. arabiensis* depends on whether its host resides indoors or outdoors. In areas where hosts mainly stay indoors, *An. arabiensis* exhibits an endophilic (indoor resting) behavioural pattern [21], whereas in areas where hosts are mainly outdoors, *An. arabiensis* exhibits both outdoor and indoor resting habits [22, 23]. The exophilic behaviour of *An. arabiensis* is also often observed following interventions such as the application of indoor residual spraying (IRS) and/or long-lasting insecticide-treated nets (LLINs) [24, 25]. A shift from endophilic behaviour to exophilic behaviour is not only seen in *An. arabiensis* but in all other malaria vector species and it is attributed to the deterrence and/or contact irritancy due to indoor malaria vector control interventions (IRS and LLINs) [25–28].

The feeding and host preference behavior of *An. arabiensis* varies considerably from place to place. Evaluation of the human blood index (HBI) of *An. arabiensis* in Ethiopia and elsewhere in Africa showed both zoophagic and anthropophagic behaviour. Fornadel et al. [29] and Tirados et al. [30] documented highly anthropophilic behavioural patterns of populations of *An. arabiensis* from Zambia and Southern Ethiopia, respectively. Similar feeding patterns of preferring humans to other non-vertebrate hosts was observed in Senegal, in a blood meal analysis of populations of *An. gambiae* and *An. arabiensis* [31]. Exclusive zoophilic behaviour of *An. arabiensis* was reported in Madagascar [32], whereas most studies on populations of *An. arabiensis* from other countries documented an opportunistic feeding behaviour [33–37].

The time of host feeding varies depending on the host preference and on whether the host stays mainly indoors or outdoors. In an assessment of hourly person-biting rates of *An. gambiae s.l.* conducted in Miwani, Kenya, a region where *An. gambiae* (54%) and *An. arabiensis* (45%) exist in sympatry, the majority (83%) of female mosquitoes were found to be biting between 01:00 and 06:00, with a peak indoor biting at 06:00, while the peak outdoor activity occurred between 02:00 and 04:00 [38]. In Ahero village, where *An. funestus* comprised a large proportion of mosquitoes caught indoors (67.3%), the main indoor biting peak for *An. arabiensis* occurred at 03:00, while the outdoor biting activity peaked between 03:00 and 06:00. The same study concluded that *An. arabiensis* mosquitoes were 1.9 times more likely to bite indoors than outdoors, and that these mosquitoes had very low preference for human blood meals as compared to *An. gambiae.* Taye et al. [39] reported that *An. arabiensis* in Southern Ethiopia bites during the entire night with a peak between 23:00 and 03:00. A recent study by Yohannes and Boelee [40] conducted in Northern Ethiopia showed that *An. arabiensis* has more early biting activities, with 70% of the biting activity occurring before 22:00, with a peak between 19:00 and 20:00, which is similar to a study conducted by Kibret et al. [41] in Central Ethiopia.

A difference in the time of biting and rhythm seems to be affected by parity, with a larger proportion of possibly disease-transmitting parous mosquitoes being active in the later part of the night, mainly when humans sleep [39, 42]. Seasonality can also influence the biting activity of populations of *An. arabiensis.* Taye et al. [39] documented that the biting rate of *An. arabiensis* in August and April were 19.3 bites/person/night and 82 bites/person/night, respectively, which is a considerable difference.

Important malaria vectors are not uniformly distributed within a country with their range typically crossing national borders. The occurrence of *Anopheles* species varies according to macro- and micro-environmental differences exhibited by different bioecological areas. Therefore, entomological studies should incorporate a detailed distribution of the vector species, as it is the basis for risk assessment of malaria transmission [43, 44]. Thus, the abundance of anophelines is one entomological parameter used to describe the relationship between vectors and the incidence of malaria [45].

One of the keystones in malaria control strategy is tackling the vector, either by reducing the vector density or infectivity rate of the vector (i.e., the proportion of sporozoite positive mosquitoes compared to the total dissected mosquitoes), which will have an impact on malaria transmission and incidence. Based on previous research reports, it appears that the vector mosquito population of Ethiopia has developed resistance against most insecticides (dichloro- diethyl-trichloroethane, permethrin, deltamethrin, and malathion) [46]. The emergence and spread of insecticide resistance in some regions may suggest that other vector control tools may be needed to sustain control and mitigate the risk of malaria infection, despite the success of existing vector control intervention strategies, such as LLINs and IRS [46]. Consequently, new attention has been given to environmental management, biological control, and zooprophylaxis [47].

In malaria vector control, zooprophylaxis can be applied separately or in combination with other vector control tools. Application of zooprophylaxis is the use of wild or domestic animals, which are not the reservoir host of a given disease, to divert the blood-seeking malaria vectors away from the human host of that disease. Use of zooprophylaxis as a malaria vector control tool can be in an active, passive, or integrated form combined with chemical insecticides used in public health [47, 48].

Research assessing the effectiveness of zooprophylaxis has been done in various countries. In this paper, a qualitative systematic review using Preferred Reporting Items for Systematic Reviews and Meta-Analyses (PRISMA) guidelines was conducted with the aim of exploring the contribution of zooprophylaxis in the fight against malaria incidence and prevalence. Therefore, we explored entomological studies of which their outcomes either favorable or non-favorable in terms of application of zooprophylaxis. Meta-analysis was not possible due to the lack of common study outcomes in the retrieved articles.

## Methods

### Identification of papers and eligibility criteria

The databases PubMed Central®, Web of Science, ScienceDirect, and African Journals Online were searched between December 2015 and March 2016. The published reports used in this review were retrieved from searches using the following key terms: “zooprophylaxis” or “cattle and malaria”, “malaria vector control”, and “host preference”. In cases where the key terms could not produce enough relevant information, references from related articles were copied and pasted in Google Scholar to get the full PDFs of the target articles. Review articles on zooprophylaxis were excluded from the synthesis but their content was assessed in order to weigh up their objective, their relevance and relatedness to our review, and their inclusiveness of contemporary information. Abstracts were selected if they were found to include information on zooprophylaxis, malaria control strategies, or on the behaviour of malaria vectors and their host preference. Irretrievable full text articles as well as non-English abstracts were excluded.

**Fig. 1:**
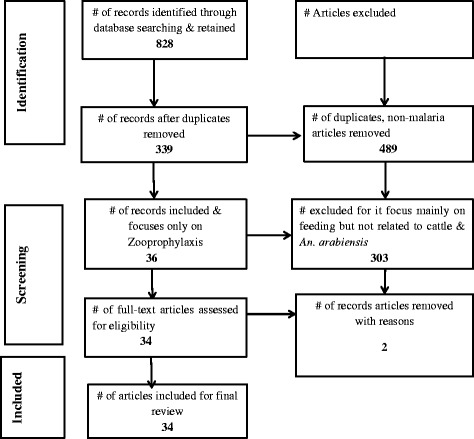
Systematic article selection

The selected articles were screened as follows: First, all abstracts not related to *Anopheles* biology, ecology, resting, feeding behaviour, feeding pattern, host preference, zooprophylaxis, or the diversion of mosquitoes to hosts other than humans were excluded. Second, duplicate and non-malaria related articles were also not considered. Bulletin news articles and articles reviewing the effects of zooprophylaxis discussed in other reviews were also excluded (see Fig. 1).

Data extraction from each article included author, date of publication, study location, mosquito species, study aim, study design, and study outcomes. Published research works reporting a significant association between the presence of livestock and reduced malaria infection were considered as supporting zooprophylaxis, and studies that either reported failure of zooprophylaxis or a poor association between zooprophylaxis and reduced malaria infection were considered as studies that disprove the effect of zooprophylaxis in malaria vector control.

## Results

### Study characteristics

Thirty-four articles were included in this review. Of these, 26 (76%) articles show that zooprophylaxis is either effective in malaria vector control, increases the incidence of malaria, or has no effect at all in malaria control. The methodologies of these articles (study aim, design, and sample size) are shown in Table 1.

**Table 1:** Summary of methodological overview (study aim, design, and sample size) of 26 studies showing that zooprophylaxis either has a positive, negative, or no effect in malaria control

Reference	Location	Study aim	Study design	Sample size
Lyimo et al., [58]	Kilombero, Tanzania	Evaluating the effectiveness of fungus bioinsecticide zooprophylaxis	Semi-field and small-scale field experimental study	1690 and 547 *An. arabiensis* from the semi-field and field, respectively, were assessed for the development of fungal infection.
Kaburi et al., [53]	Kenya	Establishing effects of zooprophylaxis and LLINs	Cross-sectional survey	80 households were surveyed; 4148 and 2615 vector mosquitoes were collected before and after the intervention, respectively, and blood sources were detected.
Bulterys et al., [49]	Zambia	Association between malaria infection and risk factors	Case-control study	34 households with malaria history in the previous two years and 37 households without malaria history in the same time period were assessed for risk factors.
Fritz et al., [59]	Kenya	Effects of ivermectin and moxidectin on malaria vectors	Laboratory-based and field-based bioassays	Exact sample size not mentioned.
Muriu et al., [54]	Kenya	To determine the blood feeding pattern of *Anopheles* mosquitoes	Longitudinal study (mosquito collection and laboratory processing)	3333 blood-fed *Anopheles* mosquitoes were collected from eight villages and blood sources were detected.
Mahande et al., [55]	Tanzania	Evaluation of feeding preference behavior	Field experimental study (mosquito collection and laboratory processing)	3902 *Anopheles* mosquitoes were collected from the field and blood sources were detected; 506 *Anopheles* were trapped using odor based entry trap (OBET) and preference was detected.
Mahande et al., [83]	Tanzania	Assessing the effect of deltamethrin-treated cattle on *An. arabiensis*	Contact bioassay and experimental hut trials	948 female *An. arabiensis* mosquitoes were used for contact bioassay.
Iwashita et al., [50]	Kenya	Assessing the added value of zooprophylaxis in the presence of ITNs	Cross-sectional survey (mosquito collection and laboratory processing, livestock survey, LLINs coverage and larval breeding habitat survey)	1664 *Anopheles* mosquitoes were examined for blood meal source and vector infection rate.
Seyoum et al., [71]	Ethiopia	To assess the impact of livestock on the HBR and malaria transmission	Longitudinal study (mosquito collection and laboratory processing, parasitological and clinical survey, field experimental tukuls trial)	Mosquitoes were collected using HLC for 12 months (once/month/3 huts) and 1180 blood samples were collected from children under 10 years of age.
Habtewold et al., [52]	Ethiopia	A blood meal analysis to determine the host preference	Cross-sectional study (mosquito collection and laboratory processing)	278 mosquitoes were tested for blood meal source and parasite positivity.
Rowland et al., [60]	Pakistan	The role of insecticide-treated livestock (dipping method) in the control of malaria	Field experimental study (Randomized controlled trial)	842 *Anopheles* mosquitoes were monitored; an average 4112 blood samples were collected and tested for parasite detection over a three-year period.
Foley et al., [61]	Indonesia	The effect of ivermectin-treated animals and humans on *An. farauti* mortality	Experimental study and modeling	Exact sample size not reported.
Hewitt and Rowland, [62]	Pakistan	The treatment of cattle with pyrethroids to control zoophilic mosquitoes	Field experimental study	38,815 anopheline mosquitoes were collected over a two-year period.
Temu et al., [64]	Mozambique	Identifying risk factors for malaria infection	Cross-sectional survey	8338 children under 15 years of age were screened for malaria detection.
Tirados et al., [70]	Ethiopia	Attraction of mosquitoes to humans in the absence and presence of cattle ring; mosquito host preference using animal and human baited traps	Field experimental study	Exact sample size not mentioned.
Yamamoto et al., [51]	Burkina Faso	The use and effects of different mosquito control measures	Case-control study	117 cases and 221 control study subjects were screened for parasites.
Githinji et al., [67]	Kenya	Interactions between humans and their micro-ecological environment	Case-control study	342 case and 328 control individuals were assessed for risk factors associated with malaria.
Deressa et al., [68]	Ethiopia	Household and socioeconomic factors associated with childhood febrile illness	Cross-sectional survey	2372 households were investigated for risk factors associated with malaria.
Tirados et al., [30]	Ethiopia	Feeding and resting preference to evaluate the protective value of cattle against *An. arabiensis*	Laboratory-based (ELISA) and Field experimental study, Longitudinal study (mosquito collection)	45,527 *An. arabiensis,* 4218 *An. pharoensis,* and 13,241 *An. funestus* group were collected
Palsson et al., [65]	Guinea Bissau	Environmental risk factors associated with increased malaria risk and vector abundance	Longitudinal study (mosquito collection)	9873 *Anopheles* mosquitoes were collected over a three-year period.
Habtewold et al., [63]	Ethiopia	Deltamethrin-treated zebu and possible behavioral avoidance of *An. arabiensis*	Contact bioassay and Field experimental study	1102 *Anopheles* mosquitoes were monitored for feeding success; 366 *Anopheles* mosquitoes were tested for blood meal source.
Bøgh et al., [57]	The Gambia	Effect of passive zooprophylaxis on malaria transmission	Paired cohort study of 102 children under age 7	A total of 204 children were monitored for malaria in the presence and absence of cattle.
Idrees and Jan, [81]	Pakistan	To determine the role of cattle ownership on the prevalence of malaria	cross-sectional survey	1873 blood samples were collected and tested for malaria.
Ghebreyesus et al., [69]	Ethiopia	Household risk factors associated with malaria incidence	Cross-sectional survey	2114 children under 10 were screened for malaria and associated risk factors.
Bouma and Rowland, [66]	Pakistan	Parasite prevalence in children housing with or without cattle	Cross-sectional survey	2042 blood samples were collected from school children aged 2–15.
Mayagaya et al., [82]	Tanzania	To investigate the impact livestock ownership has on vector ecology and malaria parasite infectivity rate	Longitudinal study (mosquito collection)	29,393 *Anopheles* mosquitoes were collected over a three-year period.

Thirteen (38%) articles show that zooprophylaxis is effective in malaria vector control. Of these, three research works were conducted in Asia (1 from Indonesia, and 2 from Pakistan), and the remaining ten were reported from Africa (nine from east Africa and one from southern Africa). Regarding the study design, there was one case-control, one laboratory-based and field-based bioassays, one contact bioassay and field experimental hut trial, one human landing catch (HLC) and parasitological survey, one randomized controlled trial, one cross-sectional, and seven experimental studies (see Tables 1 and 2).

**Table 2:** Summary of 26 studies showing outcome parameters and whether zooprophylaxis is effective or not in malaria control

Reference	Mosquito species	Outcome parameter	Percent protection	Conclusion (yes/no to zooprophylaxis; that is, is it effective or not?)
Lyimo et al., [58]	*An. arabiensis*	Mosquito mortality, fecundity	90% of mosquitoes fed on fungus-treated cattle become infected immediately and 70% of the infections occurred after three days.	Yes (if cattle treated with bioinsecticide)
Kaburi et al., [53]	*An. gambiae* complex, *An. funestus*	Man biting rate, HBI, CSP	MBR ratio decreased significantly, with RC of (−0.96; SE = 0.834; *P* = 0.017). HBI decreased significantly with RC of (0.239; SE = 0.039; *P* = 0.015 [*P* < 0.05]), especially in households with >4 cattle.	Yes (if cattle and LLINs co-applied)
Bulterys et al., [49]	*An. arabiensis*, *An. funestus*	Parasite prevalence	The risk of *P. falciparum* infection (a*OR* = 0.13; 95%*CI* = 0.03–0.56) was reduced.	Yes (if cattle sheds are separated from human quarters)
Fritz et al., [59]	*An. gambiae* s.s., *An. arabiensis*	Mosquito density, HBI	90% mortality of mosquitoes were fed on ivermectin-treated cattle.	Yes (if cattle treated with systemic insecticide)
Muriu et al., [54]	*An. arabiensis*, *An. pharoensis*, *An. funestus*	HBI, bovine blood index	71.8% indoor and 41.3% outdoor collected mosquitoes, respectively, were fed on bovine.	Yes
Mahande et al., [55]	*An. arabiensis*, *An. gambiae*	Mosquito density, HBI	90.3% of mosquitoes were trapped by cattle odor and 9.7% of mosquitoes were trapped by human odor (*P* = 0.005). A lower HBI was recorded in both the outdoor (0.1–0.3) and indoor (0.4–0.9) collected mosquitoes.	Yes (if cattle kept in human surroundings)
Mahande et al., [83]	*An. arabiensis*	Mosquito mortality, HBI,	50% of mosquitoes fed on treated cattle were knock downed 21 days after treatment. Treated cows caused higher mortality (mean = 2) as compared to untreated cows (m = 0.3).	Yes (if cattle is treated with deltamethrin every three weeks)
Iwashita et al., [50]	*An. arabiensis*, *An. gambiae* s.s, *An. funestus* s.s	Mosquito density, CSP rate	40.5% (*CI*: 36.9–44.2) of *An. arabiensis* fed on cattle, 12.0% (*CI*: 9.7–14.6) of *An. arabiensis* fed on humans; ITNs and cattle associated with decreased CSP.	Yes (if cattle co-applied with ITNs)
Seyoum et al., [71]	*An. arabiensis*, *An. pharoensis*	HBR, parasite prevalence	HBRs of *An. arabiensis* in mixed and separate cattle and without cattle were 8.45, 4.64, and 5.97, respectively. Similarly, mean parasitemia were 26.7%, 15.0%, and 23.85%, respectively.	Yes (if cattle is separated from human dwelling)
Habtewold et al., [52]	*An. arabiensis*, *An. quadriannulatus*	Mosquito density, HBI	A significantly higher proportion of mosquitoes was fed on livestock in site C compared to site A (*χ* ^2^ = 44.1, Df = 1, *P* < 0.001) or B (*χ* ^2^ = 25.9, Df = 1, *P* < 0001).	Yes (in certain areas)
Rowland et al., [60]	*An. stephensi*, *An. culicifacies*	Mosquito mortality, parasite prevalence	56% reduction in *P. falciparum* malaria; 31% reduction in *P. vivax* malaria	Yes (if cattle treated with insecticides)
Foley et al., [61]	*An. farauti*	Mosquito mortality	80–100% mortality observed in mosquitoes fed on treated cattle in the first three days after treatment.	Yes (if cattle treated with insecticides)
Hewitt and Rowland, [62]	*An. stephensi*, *An. culicifacies*	Mosquito mortality	50% reduction in longest vector survivors	Yes (if cattle treated with insecticides)
Temu et al., [64]	*An. gambiae* complex, *An. funestus*	Malaria incidence	Increased risk of malaria incidence (*OR* = 3.2; 95%*CI*: 2.1–4.9)	No
Tirados et al., [70]	*An. arabiensis, An. pharoensis*	Mosquito density,	There was no significant difference in mean *An. arabiensis* density (means = 24.8 and 37.2 mosquitoes/night, respectively; *n* = 12, *P* > 0.22) caught outdoors by HLC with or without a ring of cattle. The catch of *An. arabiensis* in human-baited traps (HBT) was 25 times greater than in cattle-baited traps (CBT) (34.0 vs. 1.3, *n* = 24; *P* < 0.001) whereas, for *An. pharoensis* there was no significant difference.	No
Bouma and Rowland, [66]	*–*	Parasitemia	Malaria prevalence (15.2%) was significantly greater among children of families which kept cattle than among those which did not (9.5%)	No
Yamamoto et al., [51]	*An. gambiae* s.l*., An. funestus*	Mosquito density, parasite prevalence	Positive correlation between donkeys and *An. gambiae* indoors (Pearson’s *r* = 0.21, *P* = 0.0002)	No
Githinji et al., [67]	*An. gambiae* s.l.	Parasitemia	53% increased risk of acquiring malaria if oxen kept in the house	No
Deressa et al., [68]	*–*	Parasitemia	Sharing house with livestock increases the risk of malaria (*OR* = 1.3, 95%*CI*: 1.1–1.6)	No
Tirados et al., [30]	*An. arabiensis*, *An. pharoensis*	Mosquito density, HBI, CSP	The HBIs for outdoor and indoor mosquitoes were 51% and 66%, respectively. CSP for *P. falciparum* and *P. vivax* were 0.3% and 0.5%, respectively. Five times more mosquitoes inside human baited trap.	No
Idrees and Jan, [81]		Parasitemia	Higher malaria burden was documented among children of families which kept cattle (11.20%) than among those which did not keep it (7.10%)	No
Ghebreyesus et al., [69]	*–*	Parasitemia	Sleeping with animals in the house was significantly associated with risk of malaria (*RR* = 1.92, 95%*CI*: 1.29–2.85)	No
Palsson et al., [65]	*An. gambiae* *Culex* *Aedus*	Mosquito density	Presence of pigs in a house was associated with increased mosquito abundance in the bedrooms of the same house.	No
Bøgh et al., [57]	*An. gambiae*, *An. arabiensis*, *An. melas*	Parasitemia	No significant differences in either the risk of parasitaemia (*OR* = 1.69, *P* = 0.26) or in high-density parasitaemia (*OR* = 0.73, *P* = 0.54).	Either (No significant differences in either the risk of parasitaemia)
Habtewold et al., [63]	*An. arabiensis*, *An. pharoensis*, *An. tenebrosus*	Mosquito mortality	Deltamethrin applied to Zebu cattle was able to provoke up to 50% mortality in mosquitoes for the 1st four consecutive weeks and then its efficacy declined after wards.	Either (reduction in density was not significant)
Mayagaya et al., [82]	*An. gambiae s.l.* *An. funestus s.l.*	Mosquito density CSP, HBI	No significance difference in mean mosquito density in households with and without livestock. Lower sporozoite rate was observed in houses with livestock however, other compounding factors should be accounted	Either

*CSP* circumsporozoite test

Another thirteen (38%) studies show that zooprophylaxis either increases the incidence of malaria or has no effect at all on malaria transmission. Of these, two research works were conducted in Asia (Pakistan), and the remaining 11 were reported from Africa (three from western Africa and eight from eastern Africa). Regarding the study design, there were three field experimental studies, one paired cohort study, two case-control studies, two longitudinal studies and the rest five were cross-sectional surveys (see Tables 1 and 2).

Eight (24%) articles are modelling studies that report the role of zooprophylaxis in malaria vector control (see Table 3).

**Table 3:** Summary of eight modeling studies that report on zooprophylaxis as a malaria vector control tool

Authors	Data source	Species	Study aim	Study design	Recommendations on the use of zooprophylaxis
Franco et al., [75]	Pakistan and Ethiopia	*An. stephensi*, *An. arabiensis*	To model the role of livestock in malaria control	Mathematical model	Livestock could have zooprophylactic effect with certain conditions such as maximum density of vector population prior to introduction, and sufficiently high number of livestock. Treatment of livestock with non-repellent insecticides and increasing the attractiveness of livestock with attractants will maximize efficacy.
Levens, [77]	Multiple sources	*An. arabiensis*	To model the role of insecticide zooprophylaxis, LLINs	Mathematical model	More than 80% coverage of LLINs to community and 80% coverage of insecticide treatment to livestock are important to achieve global reduction and elimination of the disease.
Nah et al., [76]	South Korea and others	*An. sinensis*	To investigate the effect of zooprophylaxis	Mathematical model	Decrease of animal population increases the basic reproduction number R0. Passive zooprophylaxis is an effective malaria control strategy in South Korea.
Hassanali et al., [72]	n/a	n/a	Relationship between hosts, mosquito habitat, and the relative number of individuals in the group	Computer simulation model	When the distance between human and animal host increases, the number of bites/person first decreases and is followed by an increase in the number of bites. Animals should not be placed very close to humans because it could lead zoopotnentiation and at the same animals should not be placed very far from humans otherwise they lose their protective efficacy.
Killeen and Smith, [78]	n/a	*An. arabiensis*, *An. gambiae*	To predict the effect of mass coverage of LLINs on users and non-users	Computer simulation model	With mass coverage of LLINs and IRS capable of excito-repellency in the presence of cattle, it is possible to protect both the users and non-users of ITNs.
Kawaguchi et al., [73]	n/a	n/a	Combining zooprophylaxis and IRS	Computer Simulation model	Habitat separation of cattle and humans is important for the success of zooprophylaxis. When blood host density is below the blood feeding satiation level, zooprophylaxis will fail. Spraying insecticides in human dwellings diverts mosquitoes to other hosts.
Saul, [74]	n/a	n/a	Examining the effects of animals on the transmission of vector-borne diseases	Computer simulation model	Feeding on animals decreases transmission to humans but increases mosquito survival rate. Keeping animals and humans away from breeding sites is a practical control measure. Insecticide zooprophylaxis may reduce vectorial capacity.
Killeen et al., [84]	n/a	*An. funestus*, *An. gambiae An. arabiensis*	The influence of host availability on vector blood meal choice	Computer simulation model	Increased cattle populations would cause a significant reduction in malaria in the Gambia due to a high *An. arabiensis* population, compared to no significant influence in Tanzania.

*n/a* not applicable

### Outcome parameters measured

Ten studies measure parasitaemia and/or vector abundance. Eleven studies measure mosquito abundance, HBI, and/or the sporozoite rate. Four studies measure mosquito mortality and knockdown. Two studies measure mosquito biting behaviour and human landing catch. Finally, one study uses physiological status and mosquito mortality as a response variable (see Tables 1 and 2).

### The role of zooprophylaxis in malaria control

The role of domestic animals, particularly cattle, in reducing malaria incidence differs with the zooprophylaxis type, which can be categorized as passive, active, combination, or insecticide zooprophylaxis.

Passive zooprophylaxis is the natural prophylactic effect of cattle that is seen when cattle density within a community is increased. Its effect can be studied by evaluating the association between domestic animal ownership and parasitaemia [49–51], or mosquito blood meal source, mosquito infectivity [30, 50, 52, 53] or mosquito density [35, 54, 55].

Active zooprophylaxis refers to the deliberate introduction of domestic animals in order to divert mosquitoes away from human settlements towards other non-transmitting hosts. Active zooprophylaxis is studied by evaluating the association between malaria prevalence and cattle ownership using paired cohort studies of people living with cattle placed at close proximity and people living with cattle placed at a distance [56, 57].

Combination zooprophylaxis refers to the use of insecticide treated nets (ITNs) and IRS being integrated with livestock placed in a separate shed in order to induce a push-pull effect, thereby aiming to reduce the risk of disease incidence. The deliberate introduction of LLINs and IRS is used as the pushing factor, whereas domestic animals placed strategically is used as the pulling factor. Zoophilic and opportunistic mosquitoes such as *An. arabiensis* are attracted by domestic animals, particularly cattle (i.e. pulling effect), and the chemicals used in the impregnation of bed nets and IRS are capable of inducing repellence of the vector before it comes into contact with the human host. The effect is studied by evaluating the association between ITN ownership, IRS coverage, livestock ownership, and malaria prevalence [50, 53].

Insecticide zooprophylaxis is the treatment of cattle by sponging or dipping the cattle with insecticides in order to pass on a lethal dose of insecticides to the blood-feeding mosquitoes. This effect can be studied by evaluating the difference in mosquito mortality and density, and malaria incidence in households with both treated and untreated domestic animals [56, 58–63].

The studies examined in this scoping review found that zooprophylaxis can have a positive, negative, or no effect in malaria vector control. In terms of the negative effect, pig and donkey keeping was reported to be a risk factor for malaria transmission in Mozambique [64], Guinea Bissau [65], and Burkina Faso [51]. Similarly, Bouma and Rowland [66] noticed an increased *Plasmodium* prevalence in children in Pakistan living in households with cattle, and Githinji et al. [67] concluded that the presence of cattle and long grass in homesteads results in a 1.81 higher risk for malaria infection in Kenya. Similarly, in studying the risk factors associated with malaria incidence, it was concluded that humans sleeping in the house with animals have a significantly higher risk of contracting malaria in Ethiopia [68, 69].

Several research outputs on the other hand, either lack strong conclusion with reference to the role of zooprophylaxis or the reduction in the risk of malaria infection has been attributed to other confounding factors. For instance, research conducted in the Gambia by Bøgh et al. [56, 57] suggested reduced HBI and CSP rate for mosquitoes collected from households living with cattle as compared to those mosquitoes from households without cattle. However, there was no significant difference between the groups in the HBI and CSP rates neither of *An. gambiae* s.l. nor in the estimated malaria transmission risk. Furthermore, the decrease in parasitaemia, in households living with cattle could be attributed to the fact that cattle owners were wealthier than non-cattle owners were, therefore less risk of malaria infection could be associated with improved life standard of cattle owners.

Tirados et al. [30] conducted an entomological study on *An. arabiensis* and *An. pharoensis* mosquitoes in Arba Minch, Southwestern Ethiopia in order to determine the host preference, resting behaviour of the vector population, and protective value of cattle against malaria. They concluded that cattle have a protective value against *An. pharoensis* (secondary vector) both indoors and outdoors. *An. arabiensis* (major vector) mosquitoes from this area, however, remain anthropophagic, exophagic, and exophilic, and can sufficiently feed on humans to transmit the disease. Therefore, humans staying indoors are only mildly protected if cattle are outdoors. Habtewold et al. [63] also assessed the effectiveness of deltamethrin-treated zebu and the related behavioural avoidance of *An. arabiensis* in the same region, and concluded that cattle have a protective value against *An. pharoensis.* However, no zooprophylactic effect was observed by placing zebu cattle near humans for *An. arabiensis*.

A number of reports and modelling studies argue that zooprophylaxis is effective under specific circumstances. According to Tirados et al. [70], zooprophylaxis is only effective for *An. arabiensis* when humans are indoors and cattle are outdoors. The human biting rate (HBR) was reported to be highest in mixed dwellings and lowest when cattle are kept separately both in Ethiopia [71] and Zambia [49]. This is also supported by modelling studies conduced by Hassanali et al. [72], Kawaguchi et al. [73], and Saul [74], who argue that separating the habitats of cattle and humans is necessary for the success of zooprophylaxis. This is due to the fact that the presence of cattle may decrease malaria transmission to humans but increase the mosquito survival rate. In addition to habitat separation, the animal population should increase above a threshold value, which results in the diversion of mosquitoes being a more effective malaria control strategy than decreasing the mosquito population [75, 76].

Reports confirming the effectiveness of zooprophylaxis are from African and Asian countries. Six studies are field experiments on insecticide zooprophylaxis. Regarding the successfully used treatments on cattle, these include fungus (bioinsecticide zooprophylaxis) [58], ivermectin [59, 61], deltamethrin [55, 60, 62], permethrin, and lambda cyhalothrin [62]. It was found that fungal, ivermectin, and deltamethrin-treated animals significantly reduce survival rates of malaria vectors, as well as fecundity. Residual effects are longest in deltamethrin-treated cattle. Studies on passive zooprophylaxis are mainly population-based case control studies and surveys. In these studies, different household risks for the transmission of malaria were evaluated. The combination effect of ITNs, IRS, and livestock was also assessed [50, 53, 73, 77, 78].

Deressa et al. [68], Kaburi et al. [53], and Iwashita et al. [50] collected mosquitoes from households, made inventories of livestock, and assessed the presence or absence of LLINs in Kenyan households. They found that both the person-biting rate and the HBI of *An. arabiensis* decrease with an increase in the number of cattle in households with LLINs, demonstrating the additive role of LLINs in zooprophylaxis. This is also supported by modeling studies conducted by Levens [77], and Killeen and Smith [78], who argue that scaling up mass coverage of LLINs to 80% in the community and ensuring a 80% coverage of livestock treatment with pyrethroids could lead to a global reduction and elimination of the disease.

The separation of human shelters and animal sheds at a certain distance [50–57, 66, 69] can be combined with the use of LLINs and IRS [50, 53], and the treatment of domestic animals with appropriate insecticides [55, 58–63]. The type of mosquito species and its feeding and resting behavior affect the efficacy of zooprophylaxis. Thus, ownership of domestic animals in the presence of anthropophilic vectors such as *An. gambiae* and *An. funestus* may lead to an increased risk of malaria incidence. In contrast, ownership of domestic animals may lead to a lower risk of malaria incidence in areas where zoophilic and/or opportunistic vector species such as *An. arabiensis* and *An. pharoensis* predominate [30, 50, 57, 63].

## Discussion

Malaria remains a major public health burden in Sub-Saharan Africa and continually finding effective control strategies is of great importance. For zooprophylaxis to be an effective control strategy, several conditions are required. A zoophilic and exophilic vector is the most essential component for zooprophylaxis to be effective. Habitat separation between human and host animal quarters is the second most important condition. Third, zooprophylaxis can be augmented through insecticide treatment of the animal and co-intervention with LLINs and/or IRS.

The main vectors identified that can successfully be controlled with zooprophylaxis were *An. arabiensis* and *An. pharoensis* in Africa [49, 52, 53, 55, 70, 71], and *An. stephensi*, *An. culicifacies*, *An. sinensis*, and *An. farauti* in Asia [60–62, 76].

*Anopheles arabiensis* is one of the main vectors of malaria in Sub-Saharan Africa. It is known mostly for zoophilic [32, 36, 52, 53, 55], opportunistic [35, 79], and occasionally anthropophilic behaviour [29, 30, 80]. Thus, the behaviour of *An. arabiensis* can be varied depending on the location of the host (indoor versus outdoor) and local genotype of vector population, with the West African population mostly identified as anthropophilic and the eastern counterpart being more zoophilic [30, 56]. It may therefore be concluded that *An. arabiensis* is an opportunistic feeder, feeding on both human and cattle depending on host availability. This is the basis of a line of thought that zooprophylaxis can be introduced to control malaria where *An. arabiensis* is the main malaria vector.

Separation of human living quarters and livestock quarters was found to be another key precondition in the process of implementing zooprophylaxis. In almost all instances where people and livestock shared the same house, people ended up at a higher risk of malaria infection [51, 64–67]. Thus, the presence of cattle may reduce the HBR as well as the HBI, but this is no guarantee for decreasing the estimated transmission risk or having a significant prophylactic effect. The fact that cattle may play a role as an attractant for vectors to human resting places has been proven in several reports [51, 64–70, 81, 82].

In addition to the presence of zoophilic vectors and the separation of humans and cattle, zooprophylaxis can be further strengthened if augmented with other interventions. This may include treatment of livestock with insecticides, with the primary purpose of killing mosquitoes that feed on the animal. Several reports show the success of this, including with using fungus formulations (bioinsecticide zooprophylaxis) [58], ivermectin [59, 61], deltamethrin, [60, 62, 83], permethrin, and lambda cyhalothrin [62]. In all instances, insecticide-treated animals significantly reduced survival rates of malaria vectors, as well as fecundity. Residual effects were longest in deltamethrin-treated cattle. Furthermore, a lower risk of malaria was reported when zooprophylaxis and other mainstay vector tools (LLINs and IRS) were used in combination [50, 53, 73, 77, 78].

As a negative side effect, the presence of cattle leads to a higher survival rate of *An. arabiensis* due to the abundance of available blood meals, increasing the mosquito population. This phenomenon of zoopotentiation calls for the need to evaluate zooprophylaxis as a control strategy thoroughly before introducing it into a community. Zoopotentiation may not only occur through an increase in blood meals and host availability, but also through cattle puddles, which provide an ideal breeding site for the development of mosquito larvae, hence increasing the mosquito population [74, 84].

Another point of caution is the fact that when mosquito abundance is enlarged, other vector-borne diseases may also increase in incidence. Both passive and active zooprophylaxis only divert mosquitoes to different hosts but cause no decrease in vector abundance. The advantage of insecticide zooprophylaxis is its ability to reduce the survival and fecundity of mosquitoes. However, this is not necessarily beneficial. A decrease in the number of zoophilic vectors may give rise to an increase of a different and possibly more anthropophilic vectors indirectly via decreased competition for larval space and resources. The result would be that insecticide zooprophylaxis would only reduce malaria transmission temporarily. Thus, further research on the possible consequences of the use of insecticide zooprophylaxis is required to make a more accurate evaluation. This review had certain limitations. A more objective selection of reports could be made by letting a number of people independently select or exclude certain reports. This could result in a more detailed description of the different methods used in experiments on zooprophylaxis.

## Conclusions

Zooprophylaxis should be evaluated using a site-specific approach, as in some areas it is effective whereas in others it is not. The effectiveness depends on several factors including distance from human dwelling to the breeding site of mosquitoes and the use of other control strategies such as LLINs and IRS. These factors influence the resting behaviour of local malaria vectors. Moreover, the zoophilic behaviour of *An. arabiensis* varies in different African countries, showing a more anthropophilic behaviour in West Africa as compared to countries more to the east of the continent. This suggests that zooprophylaxis could be more effective in some East African countries, where the species are zoophilic. The use of other malaria control strategies may have also influenced the evaluated results of experiments on zooprophylaxis. Future studies, such those on an estimation of the distance threshold between human quarters and livestock pens, and the additive effect of repellents on zooprophylaxis, could further strengthen the value of zooprophylaxis in malaria vector control.

## Additional file

Additional file 1: Multilingual abstract in the six official working languages of the United Nations. (PDF 609 kb)

## Abbreviations

HBI: Human blood index
HBR: Human biting rate
IRS: Indoor residual spray
ITN: Insecticide treated net
LLIN: Long-lasting insecticide-treated net
OBET: Odour based entry trap
WHO: World Health Organization
