# Supplementary material for: Zooprophylaxis as a control strategy for malaria caused by the vector Anopheles arabiensis (Diptera: Culicidae): a systematic review
Source: Infect Dis Poverty. 2017 Oct 25;6:160. doi: 10.1186/s40249-017-0366-3 (PMC5697156; doi:10.1186/s40249-017-0366-3)
Supplement: Additional file 1: — Multilingual abstract in the six official working languages of the United Nations. (PDF 609 kb) [file 40249_2017_366_MOESM1_ESM.pdf]

Translation of the abstract into the five official working languages of the United Nations

الاتقاء الحيواني كإستراتيجية مكافحة لحالات المالاريا الناجمة عن نلقلات الأمراض كبعوضة الملاريا العربية (ذوات الجناحين: البعوضيات): مراجعة منهجي

أبيب أسيل، ولوك دوشيو، وبريخت ديفليسشوير، وجيردين هسمان، وديليناساو يوهالاو

ملخص

لمحة عامة: الاتقاء الحيواني هو استخدام الحيوانات البرية أو المنزلية، التي ليست مضيفة للمرض، لإلهاء ناقلات الملاريا التي تسعى للدم من المضيفين البشريين. في هذا البحث، قمنا بمراجعة منهجية للاتقاء الحيواني لتقييم فعاليته كإستراتيجية لمكافحة الملاريا وتقييم الطرق الممكنة لتطبيقها.

الأساليب: تم البحث في قواعد البيانات الإلكترونية، وبابميد سينترال، وويب أوف سينس، وسينس ديريكت، وأفريقان جورنال أونلاين باستخدام المصطلحات الرئيسية: "الاتقاء الحيواني" أو "ماشية ومالاريا"، والتقارير المنشورة بين يناير 1995 ومارس 2016. وتم أختيار أربعة وثلاثين تقريرًا عن الاتقاء الحيواني من أجل المراجعة المنهجية.

النتائج: قد تقرر أن بعوضة الملاريا العربية هي مغذ انتهازي. فهى لديها تفضيل قوي لرائحة الماشية بالمقارنة مع رائحة الإنسان، ولكن تتغذي على كل المضيفين. يعتمد سلوكها في التغذية على المضيفات المتاحة، وتختلف من مضيفات تعيش في الداخل أو تتغذى في الداخل أو تعيش في الخارج أو تتغذى في الخارج. هناك ثلاثة عوامل أساسية للاتقاء الحيواني لتكون فعالة في الممارسة العملية: الناقلات الوالعة بالحيوان والنائية، وفصل الموائل بين الأوساط البشرية والحيوانات المضيفة، وزيادة الاتقاء الحيواني مع المعالجة بالمبيدات الحشرية للحيوانات أو التدخل المشترك من علاج الناموسيات بمبيدات الحشرات طويلة الأمد و / أو الرش في أماكن الإقامة المغلقة. ولا يمكن تطبيق الاتقاء الحيواني غير الفعال إلا في مكافحة ناقلات الملاريا إذا تم فصل مساكن الماشية والمساكن البشرية من أجل تجنب مشكلة الاتقاء الحيواني.

الاستنتاجات: تفاوتت نتائج استخدام الاتقاء الحيواني كإستراتيجية لمكافحة الملاريا في المواقع. وعليه ينصح بإجراء تقييم موقع محدد لفعاليته في مكافحة ناقلات الأمراض قبل تطبيق الاتقاء الحيواني كسلوك لـبعوض الملاريا العربي ولاختلاف البعوض عبر المناطق والأحوال.

Translated from English version into Arabic by selvana, through

TRANSLATORS
WITHOUT BORDERS

以动物预防作为由阿拉伯按蚊（***Anopheles arabiensis***）所致疟疾的控制策略：一项系统综述

Abebe Asale, Luc Duchateau, Brecht Devleesschauwer, Gerdien Huisman, and Delenasaw Yewhalaw

摘要

引言：动物预防是指利用非某特定疾病储存宿主的野生动物或家畜分散转移吸食人类宿主血液的疟疾媒介。本文统地回顾了动物预防措施，以评估其作为疟疾控制策略的有效性，并评估可能使用的方法。

方法：在电子数据库 PubMed Central®，Web of Science，Science direct 和非洲期刊在线使用关键术语“zooprophylaxis”或“牛和疟疾”进行检索，以及 1995 年 1 月至 2016 年 3 月期间发表的报告。对 34 份关于动物预防的报告进行了系统综述。

结果：结果表明，阿拉伯按蚊是机会性的吸血媒介。与人类的气味相比，它对牛的气味有强烈的偏好，但它们兼吸人血和牛血。它的摄食行为取决于周围存在的宿主，存在家栖性和户内吸血习性，以及野栖性和户外吸血习性。在实践中，动物预防的三个主要因素分别是嗜吸动物血和野栖，人和宿主动物栖息地分离，及采用杀虫剂处理动物或使用长效杀虫剂处理过的蚊帐和/或室内滞留喷洒进行共同干预以增强动物预防。在牛和人住所分开的情况下，被动的动物预防措施才能用于疟疾媒介控制，以避免动物势差的问题。

结论：在不同地点使用动物预防措施作为一种疟疾控制策略的结果有所不同。因此，建议在实施动物预防措施之前，应对其在媒介控制的有效性方面进行现场特异性评估，因为在不同地点和环境中，传疟媒介的行为各不相同。

Translated from English version into Chinese by Xin-Yu Feng

CHINESE CENTER FOR DISEASE CONTROL AND PREVENTION
NATIONAL INSTITUTE OF PARASITIC DISEASES

Зоопрофилактика как стратегия борьбы с малярией, вызванной переносчиком *Anopheles arabiensis* (Отряд: Двукрылые. Семейство: Комары): Систематический обзор

Абеб Асале, Люк Дюшато, Брехт Девлессхаувер, Гердьен Хьюисман и Деленасав Юйхало

АННОТАЦИЯ

Контекст исследования: *Зоопрофилактика - это использование диких или домашних животных, не являющихся резервуарными хозяевами заболевания, для отвлечения переносчиков малярии от человека. В данной работе мы проводили систематический обзор зоопрофилактики для оценки эффективности ее использования в качестве стратегии борьбы .с малярией и оценки возможных методов ее применения*

Методы: *Были изучены электронные базы данных "PubMed Central®", "Web of Science", Science direct" и "African Journals Online" с использованием ключевых терминов" зоопрофилактика» или «крупный рогатый скот и малярия», а также отчеты» опубликованные в период с января 1995 года по март 2016 года. Для систематического . обзора были отобраны тридцать четыре доклада по зоопрофилактике*

Результаты: *Было установлено, что Anopheles arabiensis использует оппортунистическую стратегию добывания пищи. Он предпочитает крупный рогатый скот человеку, но питается кровью и тех, и других. Его кормовое поведение определяется наличием доступных хозяев и варьируется от эндофильного и эндофагового до экзофильного и экзофагового.* Существует три основных фактора, обеспечивающих эффективное применение зоопрофилактики: наличие зоофильного и экзофильного переносчика, разделение среды обитания человека и животных, а также повышение эффективности зоопрофилактики посредством обработки животных инсектицидами или использования сеток, обработанных инсектицидами длительного действия, и/или проведения дополнительного опрыскивания инсектицидами внутренних жилых помещений. *Пассивная зоопрофилактика может применяться для борьбы с малярией, только если крупный рогатый скот и жилье человека .разделены, во избежание проблем с зоопотенциацией*

Выводы: *Результаты применения зоопрофилактики в качестве стратегии борьбы с малярией варьировались в зависимости от местности. Поэтому рекомендуется перед внедрением зоопрофилактики проводить оценку потенциальной эффективности ее использования в борьбе с переносчиками малярии на каждом конкретном участке, поскольку поведение .москитов Anopheles arabiensis зависит от характера местности и других обстоятельств*

Translated from English version into French by Suzanne Assenat, through

La zooprofilaxis como estrategia para controlar la malaria causada por el vector *Anopheles arabiensis* (Díptero: Culicidae): un análisis sistemático

Abebe Asale, Luc Duchateau, Brecht Devleesschauwer, Gerdien Huisman y Delenasaw Yewhalaw

RESUMEN

Antecedentes: La zooprofilaxis es la utilización de animales salvajes o domésticos, que no son portadores reservorio de una enfermedad determinada, para alejar de los huéspedes humanos a los vectores de malaria que buscan sangre. En este trabajo, llevamos a cabo un análisis sistemático de la zooprofilaxis, a fin evaluar su eficacia como estrategia para controlar la malaria y sus posibles métodos de aplicación.
Métodos: Se llevaron a cabo búsquedas en las bases de datos electrónicas PubMed Central®, Web of Science, Science Direct y African Journals Online, utilizando los términos clave "zooprofilaxis" o "bovino y malaria", y se tuvieron en cuenta los informes publicados entre enero de 1995 y marzo de 2016. Se seleccionaron treinta y cuatro informes sobre la zooprofilaxis el análisis sistemático.
Resultados: Se determinó que el *Anopheles arabiensis* es un alimentador oportunista. Tiene una fuerte preferencia por el olor del ganado bovino cuando se lo compara con el olor humano, pero se alimenta de ambos huéspedes. Su comportamiento alimenticio depende de los huéspedes disponibles, varía de endófilos y endófagos a exófilos y exófagos. Existen tres factores clave para que la zooprofilaxis sea eficaz en la práctica: un vector zoófilo y exófilo, la separación del hábitat humano de las dependencias de los animales portadores, y el aumento de la zooprofilaxis con un tratamiento insecticida para los animales o con la intervención coordinada de mosquiteros tratados con insecticida de larga duración y / o rociamiento residual en interiores. La zooprofilaxis pasiva sólo puede aplicarse para el control del vector de la malaria si se separa al ganado bovino de las viviendas humanas para prevenir el aumento de la transmisión de la enfermedad.
Conclusiones: Los resultados de la utilización de la zooprofilaxis como estrategia de control de la malaria variaron entre las diversas localidades. Por lo tanto, se recomienda evaluar su grado de eficacia para controlar los vectores de una forma específica a cada sitio antes de aplicar la zooprofilaxis, ya que el comportamiento del mosquito *Anopheles arabiensis* varía según las localidades y las circunstancias.

Translated from English version into Russian by Karina1207, through

La zooprophylaxie comme stratégie de lutte contre le paludisme causé par le vecteur *Anopheles arabiensis* (Diptera : Culicidae) : revue systématique

Abebe Asale, Luc Duchateau, Brecht Devleesschauwer, Gerdien Huisman et Delenasaw Yewhalaw

RÉSUMÉ

Contexte : La zooprophylaxie consiste à utiliser des animaux sauvages ou domestiques qui ne sont pas les réservoirs d'une maladie donnée, en l'occurrence le paludisme, pour détourner les vecteurs hématophages de celle-ci des hôtes humains. Le présent article est une revue systématique de la zooprophylaxie évaluant son efficacité comme stratégie de lutte contre le paludisme et ses méthodes possibles de mise en œuvre.

Méthodes : Nous avons effectué une recherche dans les bases de données électroniques, PubMed Central®, Web of Science, Science direct et African Journals Online en utilisant les mots-clés « zooprophylaxy » ou « cattle and malaria » et en retenant les rapports publiés entre janvier 1995 et mars 2016. Trente-quatre rapports sur la zooprophylaxie ont été retenus pour la revue systématique.

Résultats : Il s'avère qu'*Anopheles arabiensis* se nourrit de manière opportuniste. Il a une forte préférence pour l'odeur des bovins par rapport à celle des humains, mais se nourrit sur les deux hôtes. Son comportement alimentaire dépend des hôtes disponibles et varie d'endophile et endophage à exophile et exophage. Trois facteurs doivent être réunis pour que la zooprophylaxie soit efficace en pratique : un vecteur zoophile et exophile, la séparation des habitats des humains et des animaux hôtes et le renforcement de la zooprophylaxie à l'aide de traitements insecticides des animaux ou d'une co-intervention avec des moustiquaires traitées avec un insecticide de longue durée et/ou des pulvérisations résiduelles à l'intérieur. Afin d'éviter le problème de zoopotentialisation, la zooprophylaxie passive ne peut être employée pour la lutte contre le vecteur du paludisme que si les habitats du bétail et des humains sont séparés.

Conclusions : Les résultats de la mise en œuvre de la zooprophylaxie comme stratégie de lutte contre le paludisme varient d'un lieu à un autre. Il est donc recommandé d'évaluer spécifiquement son efficacité à contrôler le vecteur dans les conditions locales avant de la mettre en œuvre car le comportement du moustique *Anopheles arabiensis* varie selon les lieux et les circonstances.

Translated from English version into Spanish by Maria Paula Gorgone, through

TRANSLATORS WITHOUT BORDERS
